# Protective effects of *Monotheca buxifolia* fruit on renal toxicity induced by CCl_4_ in rats

**DOI:** 10.1186/s12906-016-1256-0

**Published:** 2016-08-17

**Authors:** Shumaila Jan, Muhammad Rashid Khan

**Affiliations:** Department of Biochemistry, Faculty of Biological Sciences, Quaid-i-Azam University, Islamabad, 45320 Pakistan

**Keywords:** *Monotheca buxifolia*, Antioxidant enzymes, Lipid peroxidation, DNA damages, Kidney

## Abstract

**Background:**

Oxidative stress is believed to be a root cause of various degenerative and fibrotic disorders. Dietary foods enrich in antioxidants can cure or curtail the progression of oxidative stress induced disorders. Fruit of *Monotheca buxifolia* is used locally for digestive and urinary tract disorders. We have evaluated the protective potential of the methanol extract of *M. buxifolia* (MBM) in rat exposed to carbon tetrachloride (CCl_4_) toxicity.

**Methods:**

Powder of the dried fruit of *M. buxifolia* was extracted twice with 95 % methanol to get the extract (MBM). Presence of polyphenolic constituents was detected by HPLC-DAD (High Performance Liquid Chromatography with Diode Array Detection) analysis. Sprague-Dawley male rats were divided in to six groups with six rats in each. Animals of Group I were kept control, while rats of Group II – Group V were treated intraperitoneally with 1 ml/kg body weight (bw) of CCl_4_ (30 % v/v; olive oil) 15 dosages in 30 days. Animals of Group III were orally administered silymarin (50 mg/kg bw) while Group IV and V with 200 and 400 mg/kg of MBM on next day of CCl_4_ treatment. Rats of Group VI were administered only with 400 mg/kg bw of MBM. Biochemical markers of the urine and serum were analyzed. Level of antioxidant enzymes, DNA damages lipid peroxides (TBARS), H_2_O_2_ and nitrite was assessed in renal tissues of rat. Histopathological changes in renal tissues of rat were also recorded.

**Results:**

HPLC-DAD analysis of MBM indicated the existence of gallic acid, catechin, caffeic acid and rutin. MBM administration significantly alleviated the toxic effect of CCl_4_ in rat and decreased the elevated level of RBCs, pus and epithelial cells, specific gravity, creatinine, urobilinogen, urea and albumin while increased the pH and urinary protein. Increase in the level of urobilinogen, blood urea nitrogen (BUN), urea and total bilirubin while decrease of albumin and total protein in serum was restored by the administration of MBM to CCl_4_ fed rat. Administration of MBM to CCl_4_ exposed rats significantly increased the activity level of phase I and phase II enzymes and GSH while decreased the level of TBARS, H_2_O_2_, nitrite and DNA damages in renal tissues of rat. Furthermore, histopathological alterations induced with CCl_4_ in renal tissues of rat were also diminished with the administration of MBM.

**Conclusion:**

Restoration of various parameters induced with toxic insult of CCl_4_ in rat suggests the antioxidant and repairing potential of *M. buxifolia* fruit in kidney disorders.

## Background

Medicinal plants have been used since ancient times in different medicinal systems such as Ayurvedic, Hindi and Unani in India and Pakistan. Traditional uses of medicinal plants have gained interest during recent years and a huge flurry of investigations have confirmed their therapeutic role in various ailments [1, 2]. Curative potential of the traditional medicinal plants is ensured due to the presence of worthy phytochemicals such as flavonoids, alkaloids, terpenoids and various phenolic compounds [3]. In local system of medicine extract, decoction or parts of the same plant are used to treat various disorders. The ethnobotanical use of the plant plays an important role in the evaluation and discovery of novel therapeutic agents [4].

Now it is an established fact that most of the ailments such as cardiovascular, cancer, diabetes, aging, and neuronal disorders arise due to the oxidative stress. During normal body function various free radicals including reactive oxygen species (ROS) and reactive nitrogen species (RNS) are perpetually generated as byproduct of the metabolic process. Under normal circumstances these moieties act as friends and mediate various physiological roles. However, excessive production of ROS/RNS compromised the cellular antioxidant defenses triggering a major threat to nucleic acid, proteins and lipids. The imbalance between oxidants and pro-oxidants can induce other metabolic responses to mediate the generation of secondary reactive species ultimately leading to cell death through necrosis or apoptosis.

A number of investigations have made the use of carbon tetrachloride (CCl_4_) to experimentally induce oxidative stress in various organs of rat [5, 6]. CCl_4_ is metabolized by cytochrome P_450_ enzyme family, with subsequent generation of highly toxic trichloromethyl (CCl_3_^●^) and trichloromethylperoxyl (CCl_3_OO^●^) radical that in turn induce damages by interfering with proteins, lipids and DNA. These free radicals trigger lipid peroxidation that in turn can generate more deleterious lipid peroxides, responsible for cell membrane injuries in renal tissues causing acute or chronic renal disorders. Living cells detoxify the damaging action of reactive species by antioxidant enzymes and metabolites in a professional way. The antioxidant enzymes such as catalase, peroxidase, and superoxide dismutase are the main stream enzymes to scavenge the free radicals. These enzymes are supported with scavengers and chelating agents to detoxify and block the generation of reactive oxygen species by sequestering of transition metals. In addition, there are thousands of non-enzymatic compounds derived from fruits, vegetables and medicinal plants which indirectly scavenge the free radicals. Health promoting activities of dietary foods usually involve the antioxidant effects which can be assessed by monitoring the level of various antioxidant enzymes in stress induced organisms for the development of safe medicines.

The involvement of oxidative stress in various disorders posed an interest to explore the natural sources such as medicinal plants or other dietary sources to prevent or curtail the progression leading to lethal disorders. Plants provide an alternative source to scavenge free radicals due to the presence of diverse nature of secondary metabolites. *Monotheca buxifolia* (Falc.) dcne ex. of family Sapotaceae is found in hilly areas of Northern Pakistan and Afghanistan. The plant bears small fruits locally called Gurgura used afresh and as dried. Earlier we have reported that the methanol extract of *M. buxifolia* fruit contained significant amount of total phenolic (48.54 ± 2.9 mg/g dry weight of extract) and flavonoid content (42.045 ± 3.1 mg/g of dry weight of extract). The extract also exhibited remarkable scavenging potential for the DPPH, ABTS, phosphomolybdate, superoxide and hydroxyl radicals, H_2_O_2_ scavenging and reducing power. The scavenging activities obtained for the extract showed significant association with the total phenolic and flavonoid content [7]. Other studies have also reported the strong antioxidant effects of the extracts obtained from *M. buxifolia*. Rehman et al. [8] reported that leaves of *M. buxifolia* are rich source of antioxidant metabolites. According to their studies leaves possess significant amount of total phenolic and flavonoid contents. The extract obtained from the leaves of *M. buxifolia* exhibited remarkable total antioxidant capacity, reducing power, iron chelation and superoxide radical scavenging activity [8]. Farid et al. [9] also reported strong antioxidant ability of the extract of *M. buxifolia* fruit. Antibacterial effect of the plant has also been investigated [10]. The anemic patients use its fruit to compensate the iron deficiency. In addition, fruit is taken for the treatment of urinary disorders [11] as antipyretic and vermifuge [12]. Marwat et al. [13] reported that local people use the fruit for their laxative, and digestive properties. β-sitosterol was isolated and characterized in the seeds oil of *M. buxifolia* [14]. On the basis of the potential in vitro antioxidant activities and the local use of *M. buxifolia* fruit in urinary disorders we planned to investigate the protective effects of the methanol extract against the renal toxicity induced with CCl_4_ in rat by analysis of urine and serum biomarkers, antioxidant enzymes, DNA damages and histopathogical studies of renal tissue. HPLC-DAD analysis of the extract was also carried out to evaluate the presence of various polyphenolic constituents.

## Methods

### Fruit collection of *M. buxifolia*

The fruit was collected in April 2010 from Khyber Pakhtunkhwa province of Pakistan and the plant was identified by its local name and later identified by Dr. Mir Ajab Khan, Department of Plant Sciences, Quaid-i-Azam University, Islamabad. A specimen ((#43729)) was kept at the Herbarium of the Pakistan Museum of Natural History, Islamabad. The fruits (5.0 kg fresh) of uniform size at maturity were collected and dried under shade to obtain 1.0 kg dry powder excluding the seeds. The powder was exhaustively extracted with methanol and after evaporation of the solvent in a rotary evaporator at 40 °C the crude methanol extract (MBM) was obtained. MBM was stored at 4 °C and used for various studies.

### Thin layer chromatography of MBM

An amount of 50 mg of MBM was dissolved in 1 ml of methanol (HPLC grade) Analysis for TLC was performed with silica coated aluminium plates (20 × 20 cm). For activation the silica plates were heated at 110 °C for 40 min. A volume of 10 μl of MBM and reference compounds including apigenin, isoquercetin, orientin, luteolin, rutin, hyperoside, myricetin, luteolin-7-glucoside, kaempherol, vitexin and isovitexin were dropped out to one end of the TLC plate, using a pointed capillary tube. Solvent for the spotted material (mobile phase solution, 150 ml) was prepared by mixing together water, acetic acid and butanol in a ratio of 5:4:1 and stored in a container with a covering to allow vapour circulation. After a gap of 20 min, plates were held vertically in this developing tank. Plates were taken out when mobile phase rose to the upper end, just below 1 cm from the end. Solvent front was marked immediately then the plate was subjected to drying and later sprayed with 2-APB 1 % solution. Detection of flavanoids was done by observing their characteristic colours under UV light at 365 nm. Retention factor values (RF values) have been taken using the formula;

$$ \mathrm{R}\mathrm{F} = \mathrm{Distance}\ \mathrm{the}\ \mathrm{spot}\ \mathrm{moved}/\mathrm{Distance}\ \mathrm{the}\ \mathrm{solvent}\ \mathrm{moved} $$

### High performance liquid chromatography (HPLC) analysis

Presence of polyphenolic components of the MBM were detected by the HPLC-DAD analysis. The apparatus used was of Agilent Germany and analytical column was of Sorbex RXC8 (Agilent USA) with 5 μm particle size and 25 ml capacity. Mobile phase was consisted of eluent A, (acetonitrile-methanol-water-acetic acid /5: 10: 85: 1) and eluent B (acetonitrile-methanol-acetic acid/40: 60: 1). The gradient (A: B) utilized was the following: 0–20 min (0 to 50 % B), 20–25 min (50 to 100 % B), and then isocratic 100 % B (25–40 min) at flow rate of 1 ml/min. The injection volume of the sample was 20 μl. Before the injection samples were filtered through 0.45 μm membrane filter. Among the standards gallic acid was analyzed at 230, rutin at 257 nm, catechin at 279 nm, caffeic acid at 325 nm and quercetin, myricetin, kampferol were analyzed at 368 nm [15]. Each time the column was reconditioned for 10 min before the next analysis. All samples were assayed in triplicates. Quantification was carried out by the integration of the peak using the external standard method. All chromatographic operations were carried out at an ambient temperature.

### Brine shrimp lethality assay for LD_50_ estimation

Survival assay against the MBM was carried out on the nauplii of brine shrimp (*Artemia salina*). For this purpose brine shrimp eggs were hatched in artificial sea water by dissolving 38 g/l of sea salt in distilled water [16]. A lamp was placed above the open side of the tank to attract the hatched shrimps towards wall of the tank. After 24 h the hatched shrimps matured as nauplii were used in the experiment. Stock solution of MBM (20 mg/ml) was prepared in 1 ml of propyleneglycol/Tween 80/water (4:1:4). The solution obtained was used for the two fold diluted serial solutions in salt water in the range of 0.08 – 10 mg/ml. A suspension of larvae (0.1 ml), containing 20 larvae, was added in to each vial and incubated for 24 h. The test tubes were examined after 6, 12 and 24 h and the dead larvae in each vial were counted. The death percentage was calculated for three independent experiments.

$$ \mathrm{Percentage}\ \mathrm{of}\ \mathrm{death}=\left[\frac{Total\  nauplii- Alive\  nauplii}{Total\  nauplii}\right]\times 100 $$

### Animal treatment

#### Acute toxicity studies

In order to investigate the acute toxicity of the methanol extract of the *M. buxifolia* fruit male and female Sprague-Dawley rats (150–200 g) were assorted in to eight groups having three rats in each. Administration of various dosages of the extract (50, 250, 500, 1000, 2000, 3000, 4000 mg/kg, p.o.) and saline (10 ml/kg) were given to different groups. The experiment was conducted according to the guidelines 425 advocated by the Organization for Economic Cooperation and Development (OECD, 2001) [17]. Abnormal behavior of rats as an effect of the different doses was recorded up to 6 h and the mortality rate was assessed for 14 days after each treatment. According to the results (mortality and abnormal behavior was not detected at the highest dose), 1/10 of the highest dose (400 mg/kg) along with 200 mg/kg was used in this experiment to evaluate the protective potential against the CCl_4_ induced renal toxicity in rat.

#### CCl_4_ induced toxicity studies

Sprague Dawley rats, each weighing about 150–200 g, were maintained in regular rat cages at 25–30 °C with normal 12 h light and dark cycles. The experimental proposal was endorsed (Bch#0231) by a committee of ethical issues, Quaid-i-Azam University Islamabad. Thirty six rats were equally divided into six groups with six rats in each group. Animals of Group I were treated as control. Rats of Group II–V were treated with CCl_4_ (1 ml/kg body weight i.p. 30 % v/v in olive oil) intraperitoneally. Animals of Group III were also treated with silymarin (50 mg/kg) whereas Group IV and V were additionally treated with MBM (200; 400 mg/kg body weight) orally. Group VI received only MBM at 400 mg/kg orally. The duration of the experiment was 30 days and animals received 15 dosages on alternate days. Urine samples were collected 24 h after the last treatment and stored at −70 °C for urine analysis. Under mild anesthesia of chloroform, dissection was performed ventrally. Blood samples were collected through cardiac puncture and were centrifuged at 500 × g for 15 min at 4 °C to collect the sera for estimation of biochemical parameters. The kidneys were also removed, washed (with ice cold saline to remove debris) and stored in liquid nitrogen at −70 °C for tissue homogenate tests. For performance of histopathological studies, small part of kidneys was stored with 10 % phosphate buffered formalin.

### Urine analysis

Urine samples were analyzed for pH, specific gravity, albumin, pus cells, red blood cells and epithelial cells by using standard diagnostic kits (Krenngasse 12, 8010 Graz, Australia). The urine samples were also analyzed for urobilinogen, creatinine, urea, albumin and protein with AMP diagnostics company kits (Krenngasse 12, 8010 Graz, Australia) according to the manufacturers’ instruction.

### Serum analysis

Level of urobilinogen, creatinine, urea, blood urea nitrogen (BUN), total bilirubin, albumin and total protein was assessed by the use of AMP diagnostics company kits (Krenngasse 12, 8010 Graz, Australia) according to the manufacturers’ instruction.

### Assessment of antioxidant enzymes in renal tissues

Renal tissues were homogenized and 10× homogenates was prepared by the addition of potassium phosphate buffer (pH 7.4). Further, the homogenate was centrifuged at 1500 × g for 10 min at 4 °C. The supernatant obtained was used for estimation of various antioxidant enzymes.

### Protein estimation

The estimation of soluble protein in renal tissues was carried out by a method reported earlier [18]. Briefly, 0.1 ml of the supernatant was added in 1 ml of alkaline solution and mixed thoroughly. After an incubation of 30 min the optical density of the mixture was recorded at 595 nm. The concentration of the soluble protein in renal tissue was calculated by using the standard currve of bovine serum albumin.

### Catalase (CAT) activity

For the determination of catalase activity in renal tissues, 25 μl of the supernatant was added to a mixture prepared by mixing of 100 μl of 5.9 mM H_2_O_2_ and 625 μl of 50 mM potassium phosphate buffer (pH 5.0). Disintegrated the H_2_O_2_ occurred by the presence of catalase in the supernatant and its concentration began to decline in the reaction mixture. Activity level of catalase was monitored by a decline in absorbance at 240 nm for 1 min. One unit of catalase activity stated the change in absorbance of 0.01 as units/min [19].

### Peroxidase (POD) activity

The method of Chance and Maehly [19] was followed to assess the peroxidase activity in supernatant of renal tissues. Reaction mixture was prepared by mixing of 25 μl of 20 mM guaiacol, 75 μl of 40 mM H_2_O_2_ and 625 μl of 50 mM potassium phosphate buffer (pH 5.0). The reaction was initiated by the addition of 25 μl of supernatant to the mixture. Change in absorbance of the reaction mixture was recorded at 470 nm for 1 min. One unit POD activity was deemed equivalent to change in absorbance of 0.01 as units/min.

### Superoxide dismutase (SOD) activity

In order to assess the SOD activity in renal samples method of Kakkar et al. [20] was followed. For this purpose homogenate was centrifuged at 1500 × g for 10 min and then it was centrifuged at 10,000 × g for 15 min. To a previously prepared reaction mixture (50 μl of 186 μM phenazine methosulphate and 600 μl of 0.052 mM sodium pyrophosphate buffer (pH 7.0); 150 μl of renal supernatant was added. The reaction was initiated by the addition of 100 μl of 780 μM NADH while to stop the reaction after 1 min 500 μl of glacial acetic acid was added. The quantity of chromogen formed was assessed by recording optical density at 560 nm spectrophotometrically. The enzyme activity was calculated as the concentration of enzyme required to inhibit the 50 % chromogen formation in 1 min. The results were expressed as units/mg protein.

### Quinone reductase (QR) activity

Method developed by Benson et al. [21] was adopted to assess the quinone reductase activity in renal tissues. Quinone reductase activity relies on reduction of 2,6-dichlorophenol indophenol (DCPIP). The assay system was composed of 233 μl of bovine serum albumin, 33.3 μl of 50 mM FAD, 6.6 μl of 0.1 mM NADPH, 710 μl of 25 mM Tris-HCl buffers (pH 7.4) and 33.3 μl of renal tissue supernatant. The QR activity was assessed by measuring the disappearance of DCPIP at 600 nm for 3 min at 30 s interval. Using molar extinction coefficient of 2.11 × 10^4^/M/cm, QR activity was interpreted as nM of DCPIP reduced/min/mg protein.

### Glutathione peroxidase (GSH-Px) activity

The glutathione peroxidase activity was estimated by following the protocol of Mohandas et al. [22]. GSH-Px activity was based on the oxidation of NADPH and decline in absorbance was recorded at 340 nm at 25 °C. An aliquot of 50 μl of renal supernatant was added to a mixture consisting of 50 μl of 1 mM sodium azide, 50 μl of 1 mM EDTA, 25 μl of glutathione reductase (1 unit/ml), 25 μl of 1 mM GSH, 5 μl of 0.25 mM H_2_O_2_ and 740 μl of 0.1 M sodium phosphate buffer (pH 7.4). To start the reaction 50 μl of 0.2 mM NADPH was added to the reaction mixture and optical density was recorded at 340 nm at 25 °C for 20 min. Distilled water was used as blank. By using molar coefficient of 6.23 × 10^3^/M/cm, GSH-Px activity was estimated as amount of NADPH oxidized/min/mg protein.

### Glutathione reductase (GSR) activity

The glutathione reductase activity in the renal tissues was assessed by the method of Carlberg and Mannervik [23]. GSR activity was based on the conversion of oxidized glutathione (GSSG) into reduced glutathione (GSH) at the expense of NADPH. Briefly, 50 μl of renal supernatant was added to a reaction mixture consisting of 25 μl of 1 mM oxidized glutathione (GSSG), 50 μl of 0.5 mM EDTA and 825 μl of 0.1 M sodium phosphate buffer (pH 7.6). Then an aliquot of 50 μl of 0.1 mM NADPH was added to the reaction mixture to initiate the process and decline in optical density was recorded at 340 nm at 25 °C for 20 min. Using molar extinction coefficient of 6.22 × 10^3^/M/cm, GSR activity was assessed as amount of NADPH oxidized/min/mg protein.

### Glutathione-S-transferase (GST) activity

The glutathione-S-transferase activity was based on the formation of conjugate between reduced glutathione (GSH) and 1-chloro-2,4-dinitrobenzene (CDNB) [24]. Briefly, reaction mixture was prepared by mixing of 12.5 μl of 1 mM CDNB and 720 μl of sodium phosphate buffer (pH 6.5) and allowed to incubate at 37 °C for 10 min. Later on 150 μl of the renal tissue supernatant was added to the reaction mixture. The reaction was initiated by the addition of 100 μl of 1 mM reduced glutathione and increase in absorbance was recorded 340 nm for 10 min. The blank used in this assay was composed of all the ingredients except the renal tissue supernatant and was replaced by distilled water. With the help of the molar extinction coefficient (9.6 × 10^3^/M/cm), enzymatic activity (GST) was determined and expressed as nM CDNB conjugate formed/min/mg protein.

### γ-Glutamyl transpeptidase (γ-GT) activity

The protocol of Orlowski and Meister [25] was followed to estimate the γ-glutamyl transpeptidase activity in renal tissues. The reaction assay was prepared by mixing of 250 μl of 4 mM γ-glutamyl *p*-nitroanilide, 250 μl of 40 mM glycyl glycine and 250 μl of 11 mM MgCl_2_ (prepared in 185 mM Tris HCl buffer). An aliquot of 50 μl of renal tissue supernatant was added to the reaction mixture. And after incubation for 10 min at room temperature 250 μl of trichloro acetic acid (25 %) was added to stop the reaction. Optical density of the supernatant after centrifugation at 2500 × g for 10 min was recorded at 405 nm. Using a molar extinction coefficient of 1.75 × 10^3^/M/cm, γ-GT activity was estimated as nM nitroaniline formed/min/mg protein.

### Estimation of biomolecules in renal tissues

#### Reduced glutathione (GSH) assay

The method of Jollow et al. [26] was followed for estimation of GSH concentration in the renal tissues. This method involves oxidation of GSH by sulfhydral reagent 5,5′-dithio-bis(2-nitrobenzoic acid) (DTNB) to form the yellow derivative 5′-thio-2-nitrobenzoic acid (TNB), measurable at 412 nm. Briefly, 500 μl of renal supernatant was mixed with 500 μl of sulfosalicylic acid (4 %) to carry out precipitation. After an hour of incubation at 4 °C, samples were centrifuged at 1200 × g for 20 min. Then 33 μl of supernatant obtained after centrifugation was mixed with 900 μl of 0.1 M potassium phosphate buffer (pH 7.4) and 66 μl of 100 mM DTNB. The optical density of the yellow colored complex was recorded at 412 nm and GSH activity was determined as μM GSH/g tissue.

#### Lipid peroxidation (TBARS) assay

The method developed by Iqbal et al. [27] was adopted to measure TBARS (thiobarbituric acid reactive substances) in the renal tissues. The assay system was prepared by mixing of 100 μl of renal tissue homogenate, 10 μl of 100 mM FeCl_3_, 100 μl of 100 mM ascorbic acid and 290 μl of sodium phosphate buffer (pH 7.4) and incubated for 1 h at 37 °C. The reaction was ceased by the addition of 500 μl of 10 % TCA and after the addition of 500 μl of 0.67 % TBA the tubes were placed in boiling water bath for 15 min. Then shifted on crushed ice for 5 min and allowed to centrifuge at 2500 × g for 10 min. In order to determine the amount of TBARS formed, the absorbance of the supernatant was recorded at 535 nm. With the help of the molar extinction coefficient (1.560 × 10^5^/M/cm), lipid peroxidation activity (TBARS) was expressed as TBARS formed/min/mg tissue.

#### Hydrogen peroxide (H_2_O_2_) assay

Pick and Keisari [28] protocol was followed to perform hydrogen peroxide (H_2_O_2_) assay. The oxidation of phenol red was carried out by H_2_O_2_-mediated horseradish peroxidase enzyme. Briefly, the reaction mixture was prepared by the addition of 1 ml of phenol red (0.28 nM) solution, 2.0 ml of lung tissue homogenate, 5.5 nM dextrose, 0.05 M phosphate buffer (pH 7.0). the reaction was initiated by the addition of horseradish peroxidase (8.5 units) and incubated for 60 min at 37 °C. To stop the reaction 0.01 ml of 10 N NaOH was added and centrifugation was done at 800 × g for 5–10 min. The absorbance of the sample was noted at 610 nm by using the reagent as a blank. The concentration of H_2_O_2_ was given as nM H_2_O_2_/min/mg tissue based on the standard curve of H_2_O_2_ oxidized phenol red.

#### Nitrite assay

The concentration of nitrite in renal tissue was assessed by the method of Green et al. [29] by using Griess reagent. The homogenate was treated with equal volume of ZnSO_4_ (5 %) and NaOH (0.3 M) and then centrifuged at 6400 × g for 15–20 min to obtain the protein free supernatant. A volume of 20 μl of supernatant was reacted with 1 ml of Griess reagent and the optical density of the assay mixture was recorded at 540 nm by using Griess reagent as blank.

#### DNA fragmentation assay

The DNA injuries in renal tissues were assessed by diphenylamine reaction method using the protocol of Wu et al. [30]. Kidney tissue about 100 mg was homogenized in Tris triton EDTA (TTE) solution. From homogenate 0.1 ml was taken in a tube and labelled B, was centrifuged at 200 × g at 48 °C for 10 min. The supernatant collected was labelled S and again centrifuged at 20,000 × g for 10 min at 48 °C. The intact chromatin obtained was labeled C. After addition of 1.0 ml of 25 % TCA to all tubes; B, S and C were incubated overnight at 48 °C. After centrifugation at 18,000 × g at 48 °C the precipitated DNA was recovered. To each tube 160 ml of 5 % TCA was added and heated for 15 min at 90 °C followed by the addition of 320 ml of freshly prepared DPA solution. Each tube was vortexed vigorously and incubated for 4 h at 37 °C. Optical density of the reaction assay was recorded at 600 nm. The results were presented as %fragmented DNA by using the formula:

$$ \mathrm{D}\mathrm{N}\mathrm{A}\ \mathrm{fragmentation}\ \left(\%\right)=\left[\frac{\mathrm{C}\times 100}{\mathrm{C}+\mathrm{B}}\right] $$

### Histopathological study of kidneys

Small pieces of kidney tissues were fixed in fixative sera for 3–4 h and after dehydration in ascending order of alcohol (80 %, 90 %, 100 %) were shifted in cedar wood oil. The tissues after becoming clear were embedded in paraplast. Thin slices (3–4 μm) were prepared with the help of the microtome and then after removing wax, it was stained with hematoxylin-eosin stain and examined by light microscopy.

### Statistical analysis

All parametric values were expressed as mean ± SE of six observations in each group. for each group. To determine the difference among various treatments for in vivo studies in rat one way analysis of variance was estimated by using the GraphPad Prism 5 software. Multiple comparisons among various treatments were determined using Boneferroni *post hoc* comparison test. A *P* value < 0.05 was considered significant.

## Results

### Estimation of LD_50_ and LC_50_ of MBM

**Fig. 1:**
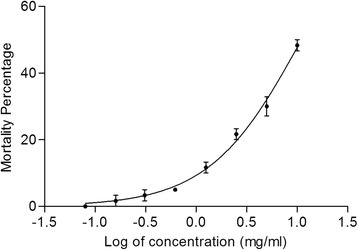
Effect of MBM on brine shrimp lethality after 24 h of treatment

The results regarding the median lethal concentration (LC_50_) of the brine shrimp lethality assay and the median lethal dose (LD_50_) of the acute oral toxicity study of MBM are presented in Table 1. The results indicated that MBM exerted very week toxicity (LC_50_ value of 4.019 mg/ml and 1.354 mg/ml) to brine shrimps at 12 h and 24 h interval of exposure (Fig. 1). However, the MBM did not exert toxicity to brine shrimps at 6 h interval of treatment. Acute oral toxicity (LD_50_) of MBM to rats indicated that the extract was not toxic even at the highest dose of 4000 mg/kg to rats.

**Table 1:** LC_50_ for brine shrimp and LD_50_ for oral toxicity studies of MBM in rat

Assays	Time	MBM
Brine Shrimp Lethality (LC_50_)	06 h	No death
	12 h	4.019 mg/ml
	24 h	1.354 mg/ml
Acute Oral Toxicity (LD_50_)	14 days	>4000 mg/kg

### HPLC-DAD studies of MBM

**Fig. 2:**
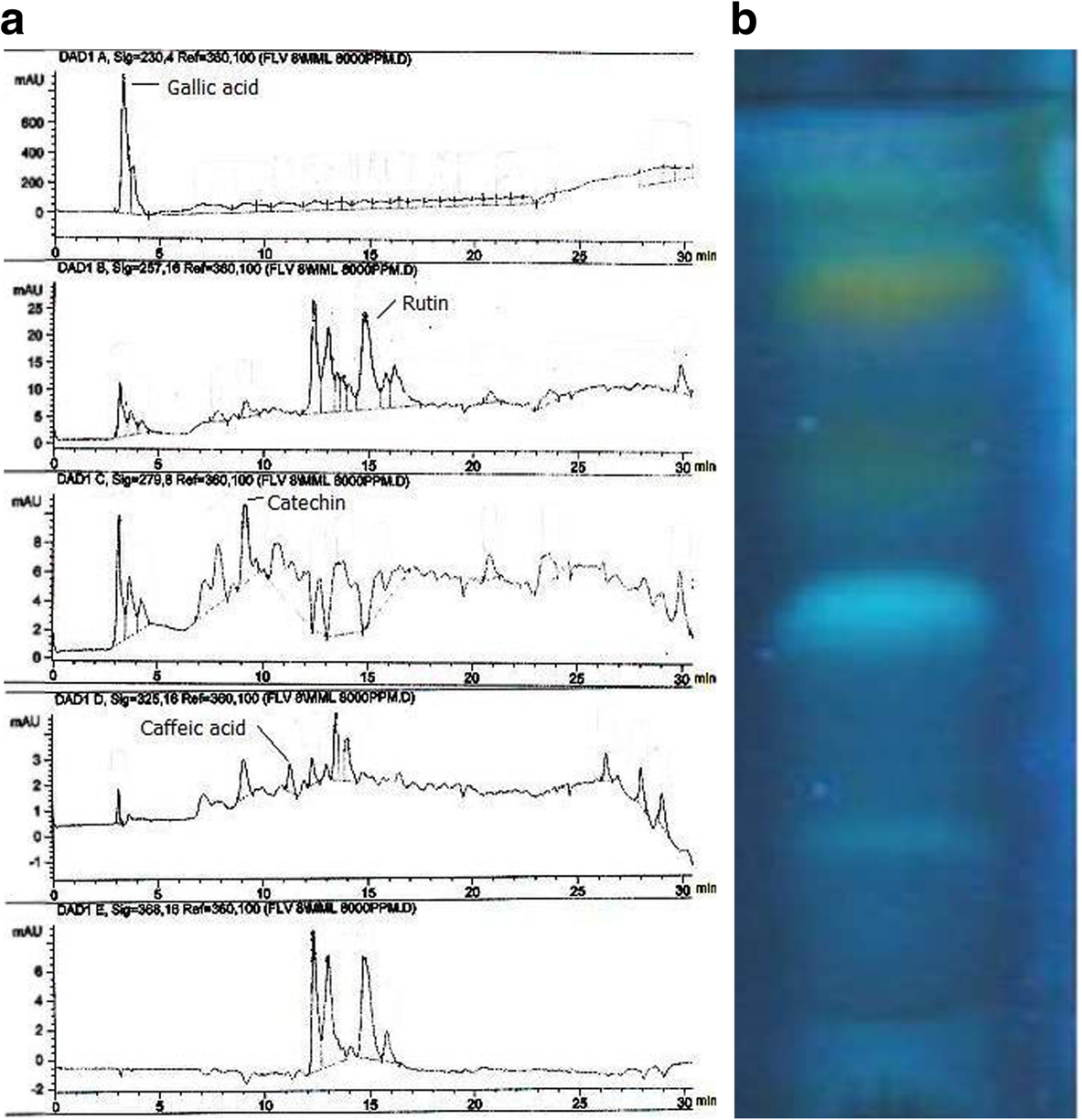
**a** HPLC-DAD analysis of methanol extract of *M. buxifolia*. **b** Qualitative separation of different polyphenols by TLC, gallic acid (R_f_: 0.21), caffiec acid (R_f_: 0.37), rutin (R_f_: 0.52) and catechin (R_f_: 0.79)

The HPLC-DAD analysis of MBM indicated the presence of gallic acid (3.329 ± 0.006 μg/mg dry extract), catechin (1.342 ± 0.004 μg/mg dry extract), caffeic acid (1.263 ± 0.003 μg/mg dry extract) and rutin (6.417 ± 0.008 μg/mg dry extract) as demonstrated in Table 2 and Fig. 2a.

**Table 2:** Polyphenolic content of methanol extract of *M. buxifolia*

Phenolic compounds	Retention Time (min)	Concentration (μg/mg dry weight)
Gallic acid	3.228	3.329 ± 0.006
Catechin	9.128	1.342 ± 0.004
Caffeic acid	11.303	1.263 ± 0.003
Rutin	14.757	6.417 ± 0.008

Values are expressed as mean ± SE (03)

### Thin layer chromatography of MBM

TLC analysis of MBM indicated the presence of gallic acid, catechin, caffeic acid and rutin. The presence of these compounds was based on the relative flow and specific color obtained under ultraviolet light of standard compounds (Fig. 2b).

### Effect of MBM on physical properties of urine in rat

Abnormalities in various urine markers were assessed in CCl_4_ treatment group and compared with those of control group (Table 3). The results indicated that pH of urine was significantly (*P* < 0.05) decreased, while specific gravity, count of RBCs, pus cells and epithelial cells was exceptionally elevated (*P* < 0.05) with administration of CCl_4_ as compared to the control group of rat. The co-administration of MBM along with CCl_4_ ameliorated the toxic effects of CCl_4_ and dose dependently reversed changes towards the control group. Treatment of the lower dose (200 mg/kg) of MBM was able to decrease (*P* > 0.05) the changes in these parameters as compared to the CCl_4_ treated group. The decreased level of pH whereas enhanced level of specific gravity, count of RBCs, pus cells and epithelial cells in urine with CCl_4_ was restored at 400 mg/kg of MBM and these parameters did not differ statistically (*P* > 0.05) from control group of rat. Similar results were found with reference drug-silymarin treated group with a trend to repair the damage caused by toxin. MBM alone treated group showed non-significant (*P* < 0.05) difference from the control for the above parameters indicating nontoxic behavior at the administered dose.

**Table 3:** Effect of *M. buxifolia* on physical properties of urine in rat

Treatment mg/kg bw	pH	Specific gravity	RBC/HPF	Pus cells/HPF	Epithelial cells
Control	7.04 ± 0.13^a^	1.05 ± 0.08^c^	1.06 ± 0.21^d^	2.41 ± 0.31^c^	1.84 ± 0.45^d^
CCl_4_	6.23 ± 0.51^c^	1.35 ± 0.09^a^	8.71 ± 1.12^a^	15.75 ± 2.36^a^	22.75 ± 2.36^a^
CCl_4_ + Sily 50	7.05 ± 0.14^a^	1.03 ± 0.09^c^	1.17 ± 0.46^c^	2.84 ± 0.82^c^	3.14 ± 0.78^c^
CCl_4_ + MBM 200	6.72 ± 0.17^b^	1.21 ± 0.12^b^	4.55 ± 0.93^b^	5.31 ± 0.55^b^	6.28 ± 1.62^b^
CCl_4_ + MBM 400	6.96 ± 0.15^a^	1.08 ± 0.07^c^	1.32 ± 0.41^c^	2.26 ± 0.67^d^	2.15 ± 0.45^d^
MBM 400	7.06 ± 0.16^a^	1.03 ± 0.08^c^	1.03 ± 0.31^d^	2.63 ± 0.74^c^	1.65 ± 0.42^d^

Values are expressed as mean ± SE (06). Means ± SE with different superscript letter ^(a–d)^ within the column indicate significant difference (*P* < 0.05 between ^a^&^b^, *P* < 0.01 between ^a^&^c^, *P* < 0.001 between ^a^&^d^). *MBM M. buxifolia* methanol extract

### Effect of MBM on biochemical markers of urine in rat

Urine analysis was done to further estimate the protective outcome of *M. buxifolia* on CCl_4_ induced changes in the urinary creatinine, urobilinogen, urea, albumin and protein in rat (Table 4). The results indicated that level of creatinine, urobilinogen, urea, and albumin was elevated while the level of urinary total protein was dropped in CCl_4_ treated group (*P* < 0.05) as compared to the control group. Administration of MBM to CCl_4_ intoxicated rats noticeably (*P* < 0.05) attenuated the toxicity of CCl_4_ on the above parameters and reversed them dose dependently towards the control group, thus improving the kidney function. MBM at its high dose (400 mg/kg) prevented the changes in the urinary creatinine, urobilinogen, urea, albumin and urinary total protein and retained the level close to the control group. Silymarin administration to CCl_4_ treated rat led to restore the level of these urine markers near the control group. Administration of MBM alone to rats did not induce changes in the aforesaid parameters in comparison to control rats.

**Table 4:** Effect of *M. buxifolia* on biochemical markers of urine in rat

Treatment mg/kg bw	Creatinine (mg/dl)	Urobilinogen (mg/dl)	Urea (mg/dl)	Albumin (mg/dl)	Protein (mg/dl)
Control	1.94 ± 0.24^d^	4.32 ± 0.48^d^	101.26 ± 3.21^d^	5.26 ± 0.44^b^	37.87 ± 1.43^d^
CCl_4_	5.13 ± 0.75^a^	33.25 ± 3.12^a^	132.45 ± 3.12^a^	19.37 ± 1.75^a^	23.94 ± 1.39^a^
CCl_4_ + Sily 50	2.65 ± 0.34^c^	11.25 ± 1.25^c^	105.28 ± 3.46^c^	7.22 ± 0.22 ^b^	34.77 ± 1.50^c^
CCl_4_ + MBM 200	3.22 ± 0.17^b^	24.21 ± 1.62^b^	114.55 ± 2.95^b^	11.42 ± 0.44^b^	26.53 ± 1.75^b^
CCl_4_ + MBM 400	2.14 ± 0.35^c^	12.68 ± 0.87^c^	106.22 ± 2.42^c^	8.45 ± 0.45^b^	34.62 ± 1.82^c^
MBM 400	2.06 ± 0.26^d^	4.23 ± 0.65^d^	103.24 ± 2.35^cd^	6.22 ± 0.32^b^	39.72 ± 1.91^d^

Values are expressed as mean ± SE (06). Means ± SE with different superscript letter ^(a–d)^ within the column indicate significant difference (*P* < 0.05 between ^a^&^b^, *P* < 0.01 between ^a^&^c^, *P* < 0.001 between ^a^&^d^). *MBM M. buxifolia* methanol extract

### Effect of MBM on biochemical markers of serum in rat

The protective effects of MBM on biochemical markers of serum in renal function are summarized in Table 5. Treatment of rats with CCl_4_ led to extensive damage to renal tissues thereby increasing (*P* < 0.05) the level of urobilinogen, urea, creatinine, BUN and total bilirubin while the concentration of albumin and total protein in serum was diminished (*P* < 0.05) as compared to the control group. Prophylactic treatment with MBM along with CCl_4_ significantly restored the altered level of above parameters. The protective effects of MBM on the amelioration of CCl_4_ induced renal toxicity were dose dependent and the level of urobilinogen, urea, creatinine, BUN and total protein in serum with 400 mg/kg dose of MBM were similar (*P* > 0.05) to the standard drug silymarin. Administration of MBM 400 mg/kg alone to rats did not indicate difference from the control in renal profile corroborating nontoxic effect of the selected dose.

**Table 5:** Effect of *M. buxifolia* on biochemical markers of serum in rat

Treatment mg/kg bw	Urobilinogen (mg/dl)	Urea (mg/dl)	Creatinine (mg/dl)	BUN (mg/dl)	Total bilirubin (mg/dl)	Albumin (mg/dl)	Protein (mg/dl)
Control	14.57 ± 1.25^d^	25.22 ± 1.29^d^	0.61 ± 0.037^d^	12.47 ± 0.85^d^	3.12 ± 0.27^c^	4.62 ± 0.26^a^	8.24 ± 0.24^a^
CCl_4_	32.84 ± 1.53^a^	75.65 ± 3.51^a^	1.82 ± 0.455^a^	35.74 ± 2.25^a^	3.92 ± 0.35^a^	2.67 ± 0.18^c^	5.65 ± 0.21^d^
CCl_4_ + Sily 50	17.75 ± 1.61^c^	29.95 ± 1.48^c^	0.95 ± 0.039^c^	16.85 ± 0.92^c^	3.25 ± 0.39^c^	3.45 ± 0.25^b^	7.34 ± 0.32^b^
CCl_4_ + MBM 200	22.53 ± 1.42^b^	39.64 ± 2.36^b^	1.28 ± 0.352^b^	22.29 ± 1.14^b^	3.52 ± 0.38^b^	3.62 ± 0.25^b^	6.67 ± 0.25^c^
CCl_4_ + MBM 400	18.50 ± 0.84^c^	28.23 ± 1.94^c^	1.04 ± 0.022^c^	15.40 ± 0.78^c^	3.24 ± 0.32^c^	4.45 ± 0.24^a^	7.43 ± 0.35^b^
MBM 400	13.45 ± 0.62^d^	26.25 ± 1.45^cd^	0.66 ± 0.026^d^	12.85 ± 0.94^d^	3.16 ± 0.22^c^	4.48 ± 0.23^a^	8.28 ± 0.46^a^

Values are expressed as mean ± SE (6). Means ± SE with different superscript letter ^(a–d)^ within the column indicate significant difference (*P* < 0.05 between ^a^&^b^, *P* < 0.01 between ^a^&^c^, *P* < 0.001 between ^a^&^d^). *Sily* silymarin, *MBM M. buxifolia* methanol extract

### Effect of MBM on phase I antioxidant enzymes in renal tissues of rat

Antioxidant enzymes are free radical scavengers that are continuously in action to protect our body from harmful effects of free radicals produced by environmental and drug toxicity. Table 6 indicated significant (*P* < 0.05) decrease in the activity of phase I antioxidant enzymes; catalase, peroxidase and superoxide dismutase in renal tissues following administration of CCl_4_ as compared to the control group. The administration of MBM to CCl_4_ induced injured rats attenuated the CCl_4_ intoxication and significantly (*P* < 0.05) restored the activity of catalase, peroxidase, superoxide dismutase and quinone reductase. Alleviation in CCl_4_ induced toxicity with MBM on the activity of antioxidant enzymes was observed in a concentration dependent manner. The activity of these enzymes in renal tissues of rat recorded with the administration of silymarin was significantly enhanced as compared to the CCl_4_ group. Administration of 400 mg/kg dose of MBM repaired the CCl_4_ induced damages and the activity level of the above enzymes in renal tissues of rats was found statistically similar to the silymarin treated group. Treatment of rats with 400 mg/kg of MBM alone did not induce toxicity in rat and the activity of catalase, peroxidase, superoxide dismutase and quinone reductase was recorded similar to the control group.

**Table 6:** Effect of *M. buxifolia* on phase I antioxidant enzyme activities in renal tissues of rat

Treatment mg/kg bw	Catalase (U/min)	POD (U/min)	SOD (U/mg protein)	QR (nM/min/mg protein)
Control	5.58 ± 0.38^a^	6.82 ± 0.46^a^	5.36 ± 0.42^a^	42.75 ± 2.03^a^
CCl_4_	2.13 ± 0.41^c^	2.98 ± 0.43^d^	1.23 ± 0.26^d^	29.95 ± 1.08^c^
CCl_4_ + Sily 50	5.25 ± 0.33^a^	5.82 ± 0.48^b^	5.14 ± 0.23^b^	39.34 ± 1.04^a^
CCl_4_ + MBM 200	3.77 ± 0.55^b^	4.72 ± 0.39^c^	2.92 ± 0.31^c^	32.52 ± 1.13^b^
CCl_4_ + MBM 400	5.13 ± 0.62^a^	5.92 ± 0.46^b^	4.96 ± 0.54^b^	39.73 ± 1.24^a^
MBM 400	5.79 ± 0.42^a^	6.62 ± 0.36^a^	5.48 ± 0.42^a^	41.24 ± 1.28^a^

Values are expressed as mean ± SE (6). Means ± SE with different superscript letter ^(a–d)^ within the column indicate significant difference (*P* < 0.05 between ^a^&^b^, *P* < 0.01 between ^a^&^c^, *P* < 0.001 between ^a^&^d^). *MBM M. buxifolia* methanol extract

### Effect of MBM on phase II antioxidant enzymes in renal tissues of rat

The activity level of phase II antioxidant enzymes; glutathione-S-transferase, γ-glutamyl transferase, glutathione peroxidase and glutathione reductase recorded in renal tissues of rat are given in Table 7. Administration of CCl_4_ to rats induced injuries in renal tissues with subsequent decrease in activity level of the glutathione enzyme family as compared to the control group. Damaging action of CCl_4_ on kidneys of was alleviated by the administration of MBM and the activity level of the above said phase II enzymes was restored towards the control group. Administration of MBM dose dependently removed the toxic effects of CCl_4_ and the activity level of these enzymes was significantly (*P* < 0.05) elevated as against the CCl_4_ treated group. Repairing potential of the standard drug silymarin on the CCl_4_ induced renal toxicity was evident and the activity level of phase II enzymes in renal tissues was statistically (*P* > 0.05) elevated to that of the CCl_4_ treated group. However, the administration of MBM 400 mg/kg dose alone did not induce statistical (*P* > 0.05) change in the activity level of the phase II enzymes of renal tissues as compared to the control group.

**Table 7:** Effect of *M. buxifolia* on phase II antioxidant enzyme activities in renal tissues of rat

Treatment mg/kg bw	GST (nM/min/mg protein)	γ-GT (nM/min/mg protein)	GSH-Px (nM/min/mg protein)	GSR (μg/mg protein)
Control	23.68 ± 1.25^a^	312.32 ± 5.00^a^	86.55 ± 2.29^d^	141.64 ± 3.93^d^
CCl_4_	9.68 ± 0.95^c^	87.54 ± 4.58^d^	35.61 ± 1.78^a^	62.55 ± 2.37^a^
CCl_4_ + Sily 50	22.65 ± 1.14^a^	303.28 ± 5.25^a^	84.33 ± 2.54^d^	137.41 ± 3.65^d^
CCl_4_ + MBM 200	15.45 ± 0.85^b^	264.42 ± 5.28^c^	57.26 ± 1.39^b^	101.39 ± 2.07^b^
CCl_4_ + MBM 400	21.73 ± 1.12 ^a^	306.24 ± 5.18^a^	79.84 ± 2.33^c^	132.22 ± 3.53^c^
MBM 400	21.62 ± 1.26^a^	309.28 ± 5.23^a^	88.53 ± 2.58^d^	145.62 ± 3.84^d^

Values are expressed as mean ± SE (6). Means ± SE with different superscript letter ^(a–d)^ within the column indicate significant difference (*P* < 0.05 between ^a^&^b^, *P* < 0.01 between ^a^&^c^, *P* < 0.001 between ^a^&^d^). *MBM M. buxifolia* methanol extract

### Effect of MBM on the biomolecules of renal tissues

Oxidative stress has been considered as one the primary reason of CCl_4_ induced injuries in experimental organisms (Table 8). Aside from the toxic effect of CCl_4_ on the antioxidant enzymes, changes in the concentration of other biomolecules have been manifested. Administration of CCl_4_ to rats significantly (*P* < 0.05) enhanced the concentration of lipid peroxides (TBARS), H_2_O_2_ and nitrite along with elevated (*P* < 0.05) injuries in DNA in renal tissues of rat as compared to the control group. Level of reduced glutathione in the kidney tissues of rat was decreased (*P* < 0.05) with the treatment of CCl_4_. Co-administration of MBM to CCl_4_ fed rats diminished the toxic effect of CCl_4_ with subsequent decrease in the level of TBARS, H_2_O_2_, nitrite and DNA injuries while increase in GSH concentration was recorded in the renal tissues of rat. The protective potential of silymarin for the above parameters was recorded similar to the higher dose (400 mg/kg) of MBM. Administration of MBM 400 mg/kg alone to rats did not alter the level of above parameters as compared to the control group.

**Table 8:** Effect of *M. buxifolia* on biomolecules in renal tissues of rat

Treatment mg/kg bw	GSH (μM/g tissue)	TBARS (nM/min/mg protein)	H_2_O_2_ (μM/ml)	Nitrite (μM/mg protein)	% DNA injuries
Control	32.67 ± 1.22^a^	3.18 ± 0.62^c^	1.32 ± 0.27^c^	105.42 ± 2.10^c^	8.21 ± 0.53^c^
CCl_4_	15.30 ± 1.37^d^	5.32 ± 0.55^a^	2.72 ± 0.36^a^	142.25 ± 2.24^a^	26.32 ± 1.29^a^
CCl_4_ + Sily 50	31.21 ± 1.49^b^	3.26 ± 0.12^c^	1.45 ± 0.24^c^	109.52 ± 2.12^c^	9.15 ± 0.70^c^
CCl_4_ + MBM 200	23.64 ± 2.53^c^	4.24 ± 0.39^b^	2.04 ± 0.35^b^	124.27 ± 2.41^b^	14.23 ± 0.85^b^
CCl_4_ + MBM 400	30.57 ± 2.32^b^	3.22 ± 0.35^c^	1.38 ± 0.24^c^	108.75 ± 2.12^c^	9.49 ± 0.72^c^
MBM 400	34.46 ± 2.94^a^	3.12 ± 0.31^c^	1.43 ± 0.23^c^	107.24 ± 2.35^c^	8.02 ± 0.37^c^

Values are expressed as mean ± SEM (06). Means ± SEM with different superscript letter ^(a–d)^ within the column indicate significant difference (*P* < 0.05 between ^a^&^b^, *P* < 0.01 between ^a^&^c^, *P* < 0.001 between ^a^&^d^), *MBM M. buxifolia* methanol extract

### Effect of MBM on renal histoarchitecture

**Fig. 3:**
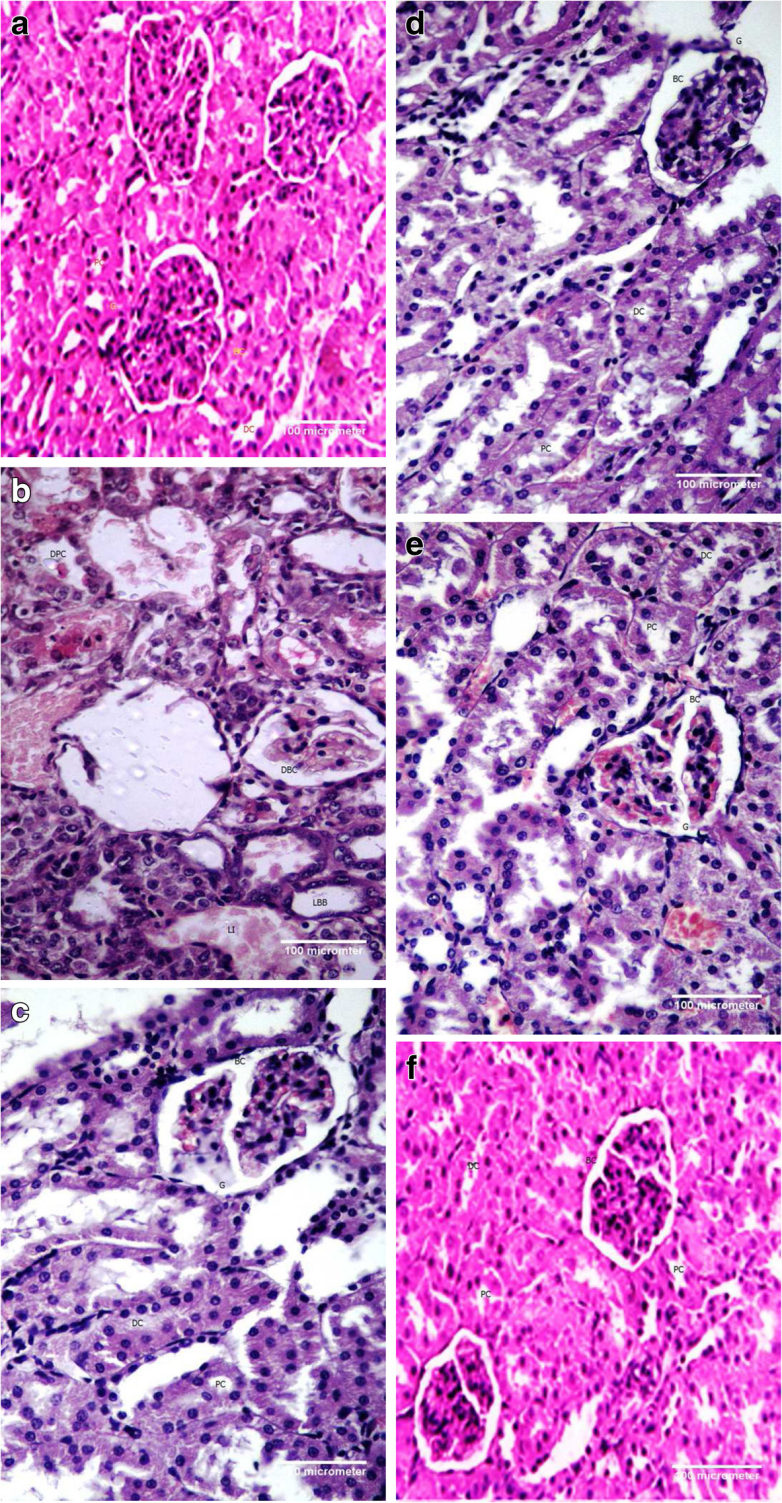
Histopathology of renal tissues (40× magnification). **a** Control group; **b** CCl_4_ treated group; **c** CCl_4_ + Silymarin (50 mg/kg bw) treated group; **d** CCl_4_ + MBM (200 mg/kg bw) treated group; **e** CCl_4_ + MBM (400 mg/kg bw) treated group; **f** MBM (400 mg/kg bw) treated group. BC; Bowmans’ capsule, G; Glomerulus, PC; Proximal Convoluted Tubule, DC; Distal Convoluted Tubule, LI; Leukocytes Infiltration, LBB; Loss of Brush Border, DBC; Damaged Bowmans’ Capsule, DPC; Damaged Proximal Convoluted Tubule

Modifications in histopathology of renal tissue are summarized in Fig. 3. Regular histological architecture was found in the control group (Fig. 3a). In the control group normal Bowman’s capsule and glomeruli with normal distal and convoluted tubules were observed. Severe histopathalogical deviations were detected in the CCl_4_ treated group (Fig. 3b). Glomeruli and Bowman’s capsule were showing abnormal appearance with diminished corticular sections and buildup of necrotic cells in CCl_4_ treated group. There were also congestion of blood capillaries, dilation of tubules, narrow30ing of Bowman’s capsule space and infiltration of inflammatory cells in medullary and corticular sections in CCl_4_ intoxicated renal tissues of rats. Treatment with silymarin and MBM reversed these pathological manifestations and tends to repair the damages which were induced by CCl_4_ treatment (Fig. 3c-e). MBM mitigated the glomerular degeneration and inflammatory cells infiltration caused by CCl_4_ (Fig. 3d). Bowman’s capsule recovered the normal space and there was no congestion of blood capillaries in glomerular portions. These drastic changes were reversed dose dependently by MBM administration (Fig. 3e). MBM alone treated group was also observed with normal renal histoarchitecture (Fig. 3f).

## Discussion

The estimation of LC_50_ for plant extract is a valuable criterion to demonstrate the presence of toxic constituents. The results obtained for LC_50_ of MBM on brine shrimp lethality assay exerted that the extract possesses bioactive compounds with very week cytotoxic effects. Previously significant correlation has been reported between the LC_50_ of brine shrimp lethality and the LD_50_ for oral acute toxicity in mice [31]. Accordingly LC_50_ < 10 μg/ml possesses LD_50_ between 100 and 1000 mg/kg; LC _50_ < 20 μg/ml possesses LD_50_ between 1000 and 2500 mg/kg, and LC_50_ > 25 μg/ml possesses LD _50_ between 2500 and 8000 mg/kg. The LC_50_ obtained at 24 h of the treatment of MBM for brine shrimps demonstrated that LD_50_ for oral acute toxicity study should be higher from 4000 mg/kg treatment.

Free radicals are classified as extremely reactive species and are considered to be implicated in varied human diseases including the chronic and acute renal disorders. These are recognized as the main factors involved in DNA damages, lipid peroxidation and protein injuries. Kidneys are unambiguously vulnerable to chemical toxicity and kidney failure is one of the dominant processes that lead towards death of the organism. Administration of CCl_4_ shifts the oxidant-antioxidant balance towards negative by disturbing the antioxidant enzyme defense system. Some studies have proved that nephrotoxic effects of CCl_4_ are associated with free radical production [5, 32]. The increase in concentration of lipid peroxides and nitrite after CCl_4_ induced toxicity in the renal tissues of rats has been demonstrated in various studies. The toxic effects of CCl_4_ and other oxidant can be minimized by the use of diets rich in antioxidant agents that have great concern to defend the biological system against oxidative damages. In this study we have assessed the presence of various polyphenolics by HPLC-DAD analysis and TLC studies in MBM. Present study showed the presence of dietary polyphenolics such as gallic acid, catechin, caffeic acid and rutin with known antioxidant activity in MBM [33, 34]. Hence, it can be proposed that *M. buxifolia* might possess protective abilities against the degenerative disorders induced by oxidative stress.

Many studies have documented that the analysis of urine profile provide enormous evidence for the physiological integrity of the kidneys. Altered level in urine profile indicated the distressed condition of kidneys. Urinary specific gravity is associated with the concentration of excretory substances. The presence of RBCs, pus cells and epithelial cells in urine are the indicator of glomerular injuries. In the present study, marked fluctuations were noticed from the normal physiological levels in urine profile of CCl_4_ treated rats. These changes might be due to the injury of the renal tissue induced by free radicals produced as a result of the metabolism of CCl_4_. According to the present investigation, CCl_4_ intoxication considerably decreased the pH, whereas specific gravity, pus cells, RBCs, albumin, and epithelial cells were found to rise as compared to control group. Alteration in these parameters after CCl_4_ intoxication provides information regarding the kidney and hepatic function and the acid base balance in the body. Under ordinary conditions urobilinogen is usually very little or no excreted into urine. Higher level of specific gravity of urine reflects the renal damages such as renal artery steatosis, decreased blood flow to kidneys, dehydration and proteinuria. The higher level of haematuria, proteinuria and urobilinogen excretion in this study might reflect the renal injuries induced with CCl_4_ administration in rat. However, ameliorating effects of MBM on the urine profile were exposed and these changes were reversed in the groups treated with MBM in a dose dependent manner. Our study is in accordance with other investigations where reversal of renal parameters towards the control level with plant extract have been reported [5, 35].

In addition to abnormal urine profile, serum profile also exhibited impaired-renal function with certain altered values in CCl_4_ intoxicated group as compared to control group. CCl_4_ intoxication to rats was accompanied with a marked increase in urobilinogen, urea, BUN, creatinine and total bilirubin levels while albumin and protein were found to decrease substantially in serum while indicating impaired kidney and/ or renal function [36]. The creatinine level in serum does not rise until severe damage occurred in the renal tissues. Abnormally high level of proteinuria in this study might be contributed by the injured nephrons. Low molecular proteins once filtered reabsorbed and metabolized by the distal portion of tubules. CCl_4_ can induce oxidative stress in different organs that provides a link between the low synthesis of protein from hepatocytes and enhanced excretion from urine. Similarly hepatocyte dysfunction can elevate the creatinine in serum. The findings are in accordance with other studies [5, 37] who reported significantly (*P* < 0.01) increase in the level of urobilinogen, urea, creatinine, BUN and total bilirubin when rats were treated with CCl_4_ intraperitoneally in contrast to the control group. However, the alterations in serum profile were diminished with MBM treatment probably by the protective potential against the CCl_4_ induced renal injuries.

Kidney is one of those main organs in which CCl_4_ causes cellular injuries by producing highly deleterious free radicals and the situation become even worse by the local endemic conditions in renal tissues. In this study level of nitrites and H_2_O_2_ was increased in renal tissues by the treatment of CCl_4_ to rats. The increase in nitrite content of renal tissues might be attributed by the oxidative stress that mediates the metabolic responses involved in vasoconstriction. A number of studies have indicated the enhanced synthesis of nitrite in the liver and kidney tissues and excretion into urine [5, 36, 38]. Development of acidic conditions as damaging action of CCl_4_ in various tissues might contribute towards the generation of nitric oxide from nitrite. Presence of superoxide in the area converts the nitric oxide into peroxynitrite; a highly damaging molecule involved in lipid peroxidation and tissue degeneration. Co-administration of MBM attenuated the toxic insult of CCl_4_ and decreased the nitrite content in renal tissues. Similar protective effects of the extracts from plants have also been reported in various studies [5, 36, 38].

Among the cellular defense system phase I and phase II antioxidant enzymes are the key players to eliminate the induced oxidative stress. The increased concentration of H_2_O_2_ in the renal tissues may be liable for the oxidative stress induced with CCl_4_ intoxication. Superoxide dismutase is mainly involved in the catalytic dismutation of highly toxic superoxide radicals to H_2_O_2_. Catalase authoritatively decomposes the H_2_O_2_ into water and oxygen in peroxisomes. Administration of CCl_4_ induces oxidative stress in renal tissues via accumulation of superoxide and H_2_O_2_ that led to a decreased level of phase I antioxidant enzymes. The results obtained with co-administration of MBM to CCl_4_ fed rats define the repairing effects towards the renal injuries induced with CCl_4_. The protective effects obtained with 200 mg/kg of MBM were more pronounced and have ameliorated the oxidative damage with subsequent elevation in the activity level of superoxide dismutase, peroxidase, catalase and quinone reductase. Administration of MBM alone did not induce any toxic effect on the phase I antioxidant enzymes of kidneys. Plant extract having dietary polyphenols exert the similar protective potential against CCl_4_ induced renal toxicity in rat [39].

In this investigation the activity level of phase II antioxidant enzymes; GST, GSR, GSH-Px and γ-GT in renal tissues was decreased by CCl_4_ administration to rats, reflecting the oxidative damage to kidney tissues. The enhanced generation of lipid peroxides through CCl_4_ toxicity lowered the activity of these enzymes that was accompanied by the lowered concentration of GSH [40]. Glutathione peroxidase removes hydrogen peroxide and other organic hydroperoxides from the environment with the support of GSH. In doing so, glutathione is oxidized which is reverted to its reduced form again by glutathione reductase. Glutathione-S-transferase plays a vital role by conjugating some potentially toxic compounds with glutathione. The free radicals which are behind the pathogenesis of renal and other forms of tissue injury involve the superoxide anion, hydrogen peroxide and the hydroxyl radicals. Last but not the least, nitric oxide and the peroxynitrite anion are also identified for their oxidant potential. CCl_4_ either directly by binding with thiol proteins and/ or indirectly via metabolites reduces the concentration of GSH in the cell [40]. These results support the view that CCl_4_ induces potential nephrotoxic effects by the depletion of GSH and consequently the activity of phase II antioxidant enzymes are being compromised. Co-administration of MBM to rats ameliorated the nephrotoxicity induced with CCl_4_ as evidenced by a decline in oxidative stress that led to significant increase in the activity of antioxidant enzymes. Restoration of GSH and phase II antioxidant enzymes in renal tissues signify that MBM act as antioxidant agent; alleviated the damaging effects of CCl_4_ and maintained the functional integrity of kidneys. Similar observations were also reported where the activity of phase II antioxidant enzymes in renal tissues was increased by plant extract in CCl_4_ intoxicated rats [5, 39].

The decreased status of antioxidant enzymes and GSH in renal tissues of CCl_4_ treated group reflects the enhanced generation of reactive species. It is conceivable that compromised activity of natural defenses initiates the deleterious process of lipid peroxidation, a remarkable pathological mechanism of renal toxicity. In this study the level of lipid peroxides (TBARS) was significantly increased in renal tissues of CCl_4_ treated rats. Decreased scavenging of reactive species in kidneys of CCl_4_ treated rats might trigger the synthesis of secondary reactive species that in turn augment the process of lipid peroxidation. Fruit of *M. buxifolia* are used traditionally to treat the urinary disorders. Administration of MBM to CCl_4_ intoxicated rats significantly diminished the level of TBARS in renal tissues, might indicated the scavenging and conversion of free radicals into non-reactive products [35].

In this study administration of CCl_4_ to rats elevated the DNA damages in renal tissues of rat. It is generally accepted that persistent generation of reactive species in a cell are involved in the formation of lipid peroxide radicals which trigger the breakdown of strand breaks and induces chromosomal mutations [41–43]. Increased level of oxidative stress enhanced the activity of Deoxyribonuclease I (DNase I) and also induces DNA damages by modifying base pairs [43]. On the other hand co-administration of MBM dose dependently decreased the DNA damages in renal tissues of rat suggesting the scavenging of reactive species and possibly by lowering the activity of DNase I. The results obtained in the present investigation are corroborated with previous findings where fruit extract decreased the DNA damages in renal tissues [5].

Histopathological investigation endorses the outcome of the biochemical studies. Histological studies revealed the marked alterations of the various treatment recipes applied to the tested animals. In the present study, the histoarchitecture of the rat kidney treated with CCl_4_ exhibited vacuolization, loss of brush borders, marked dilatation of renal tubules along with increased space between Bowman’s capsule and glomerulus. This may be due to the destruction of tissue provoked as a consequence of free radical generation via CCl_4_ metabolism. Our observations relate with previous studies who demonstrated classical damage in the rat kidney after CCl_4_ injection, as by glomerular congestion and swelling, vacuolization of convoluted tubules, atrophy of epithelial cells, pyknotic nuclei, shedding of atypical cytoplasm and loss of brush boarder [39]. In addition, lumen was also widened and marked vascular congestion was noted. In the existing study, post treatment effects of MBM maintained the normal anatomical features of the renal tissue in a dose dependent manner. Our study coincides with that of Khan and Siddique [39] who recorded that the methanol extract of *Citharexylum spinosum* cause significant recovery of renal toxicity induced with CCl_4_ in a dose dependent manner. They also narrated that polyphenolics are the phytochemicals which were responsible for therapeutic cum antioxidant action of the plant extract.

## Conclusion

In vivo assessment revealed that CCl_4_ cause deleterious effects directly by targeting lipids, DNA and proteins *via* free radical generation thus leading towards nephrotoxicity. The co-administration of MBM ameliorated the oxidative stress by normalizing the enzymatic and non-enzymatic antioxidant profile, urine and serum markers and histological architecture of kidney. So this study accredits the nephron-protective effect of *M. buxifolia* fruit probably by its polyphenolic constituents.

## Abbreviations

ABTS, 2, 2 azobis(3ethylbenzothiozoline-6-sulphonic acid; b.w, body weight; BUN, blood urea nitrogen; CAT, catalase; CCl_4_, carbon tetrachloride; DNA, deoxyribonucleic acid; DPPH, 1, 1-diphenyl-2-picryl-hydrazyl; GSH, glutathione; GSH-Px, glutathione peroxidase; GSR, glutathion reductase; GST, glutathione-*S*-transferase; H_2_O_2_, hydrogen peroxide; HPLC-DAD, High Performance Liquid Chromatography with Diode Array Detection; LD_50_, median lethal dose; MBM, the methanol extract of *Monotheca buxifolia*; NADH, nicotinamide adinine dinucleotide phosphate reduced; NaOH, sodium hydroxide; POD, peroxidase; RBC, red blood cells; RF, retention factor; RNS, reactive nitrogen species; ROS, reactive oxygen species; SE, standard error; SOD, super oxide dismutase; TBARS, thiobarbituric acid reactive substances; TCA, trichloroacetic acid; TLC, thin layer chromatography; UV, ultra violet; ZnSO_4_, zinc sulphate; γ-GT, gamma glutamyl transaminase

## References

[1] Afsar T, Khan MR, Razak S, Ullah S, Mirza B (2015). Antipyretic, anti-inflammatory and analgesic activity of *Acacia hydaspica* R. Parker and its phytochemical analysis. BMC Complement Altern Med.

[2] Bokhari J, Khan MR (2015). Evaluation of anti-asthmatic and antioxidant potential of *Boerhavia procumbens* in toluene diisocyanate (TDI) treated rats. J Ethnopharmacol.

[3] Shah NA, Khan MR, Naz K, Khan MA (2014). Antioxidant potential, DNA protection, and HPLC-DAD analysis of neglected medicinal *Jurinea dolomiaea* roots. Biomed Res Int.

[4] Ullah S, Khan MR, Shah NA, Shah SA, Majid M, Farooq MA (2014). Ethnomedicinal plants use value in the District Lakki Marwat of Pakistan. J Ethnopharmacol.

[5] Khan MR, Rizvi W, Khan GN, Khan RA, Shaheen S (2009). Carbon tetrachloride induced nephrotoxicity in rat: protective role of *Digera muricata* (L.) Mart. J Ethnopharmacol.

[6] Naz K, Khan MR, Shah NA, Sattar S, Noureen F, Awan ML (2014). *Pistacia chinensis*: a potent ameliorator of CCl_4_ induced lung and thyroid toxicity in rat model. Biomed Res Int.

[7] Jan S, Khan MR, Rashid U, Bokhari J (2013). Assessment of antioxidant potential, total phenolics and flavonoids of different fractions of *Monotheca buxifolia* fruit. Osong Public Health Res Perspect.

[8] Rehman J, Khan IU, Farid S, Kamal S, Aslam N (2013). Phytochemical screening and evaluation of in vitro antioxidant potential of *Monotheca buxifolia*. E3 J Biotechnol Pharm Res.

[9] Farid S, Khan IU, Rehman J. *In-vitro* evaluation of antioxidant activity of *Monotheca buxifolia:* A spectrophotometric approach. Amazon: LAP LAMBERT Academic Publishing; 2012. p. 1–80.

[10] Hazrat A, Nisar M, Zaman S (2013). Antibacterial activities of sixteen species of medicinal plants reported from Dir Kohistan Valley KPK, Pakistan. Pak J Bot.

[11] Murad W, Azizullah A, Adnan M, Tariq A, Khan KU, Waheed S, Ahmad A (2013). Ethnobotanical assessment of plant resources of Banda Daud Shah, District Karak, Pakistan. J Ethnobiol Ethnomed.

[12] Barkatullah, Ibrar M, Rauf A, Hadda TB, Mubarak MS, Patel S (2015). Quantitative ethnobotanical survey of medicinal flora thriving in Malakand Pass Hills, Khyber Pakhtunkhwa, Pakistan. J Ethnopharmacol.

[13] Marwat SA, Rehman FU, Usman K, Khakwani AA, Ghulam S, Anwar N, Sadiq M, Khan SJ (2011). Medico-ethnobotanical studies of edible wild fruit plants species from the flora of north western Pakistan (D. I. Khan district). J Med Plant Res.

[14] Nazir M, Rehman JU, Khan SA, Bhatty MK (1986). The constituents of unsaponifiable from *Monotheca buxifolia* seed oil. Eur J Life Sci Technol.

[15] Zu Y, Li C, Fu Y, Zhao C (2006). Simultaneous determination of catechin, rutin, quercetin, kaempferol and isorhamnetin in the extract of sea buckthorn (*Hippophae rhamnoides* L.) leaves by RP-HPLC with DAD. J Pharm Biomed.

[16] Qaiser F, Trembley JH, Kren BT, Wu JJ, Naveed AK, Ahmed K (2014). Protein kinase CK2 inhibition induces cell death via early impact on mitochondrial function. J Cell Biochem.

[17] OECD (2001). OECD guideline for testing chemicals 425. Acute oral toxicity-up and down procedure.

[18] Lowry OH, Rosebrough NJ, Farr AL, Randall RJ (1951). Protein measurement with Folin phenol reagent. J Biol Chem.

[19] Chance B, Maehly AC (1955). Assay of catalase and peroxidase. Methods Enzymol.

[20] Kakkar P, Das B, Viswanathan PN (1984). A modified spectrophotometric assay of superoxide dismutase. Indian J Biochem Biophys.

[21] Benson AM, Hunkeler MJ, Talalay P (1990). Increase of NADPH, quinone reductase activity by dietary antioxidant: possible role in protection against carcinogenesis and toxicity. Proc Natl Acad Sci U S A.

[22] Mohandas J, Marshal JJ, Duggin GG, Horvath JS, Tiller DJ (1984). Differential distribution of glutathione and glutathione-related enzymes in rabbit kidney. Possible implications in analgesic nephropathy. Biochem Pharmacol.

[23] Carlberg I, Mannervik EB (1975). Glutathione level in rat brain. J Biol Chem.

[24] Habig WH, Pabst MJ, Jakoby WB (1974). Glutathione-S-transferases: the first enzymatic step in mercapturic acid formation. J Biol Chem.

[25] Orlowaski M, Miester A (1973). γ-Glutamyl cyclotransferase distribution, isozymic forms, and specificity. J Biol Chem.

[26] Jollow DJ, Mitchell JR, Zampaglione N, Gillete JR (1974). Bromobenzene induced liver necrosis. Protective role of glutathione and evidence for 3, 4-bromobenzene oxide as a hepatotoxic metabolite. Pharmacology.

[27] Iqbal M, Sharma SD, Zadeh HR, Hasan N, Abdulla M, Athar M (1996). Glutathione metabolizing enzymes and oxidative stress in ferric nitrilotriacetate (Fe-NTA) mediated hepatic injury. Redox Rep.

[28] Pick A, Keisari Y (1981). Superoxide anion and hydrogen peroxide production by chemically elicited peritoneal macrophages-induction by multiple non phagocytic stimuli. Cell Immunol.

[29] Green LC, Wagner DA, Glogowski J, Skipper PL, Wishnok JS, Tannenbaum SR (1982). Analysis of nitrate, nitrite, and [^15^N] nitrate in biological fluids. Anal Biochem.

[30] Wu B, Ootani A, Iwakiri R, Sakata Y, Fujise T, Amemori S, Yokoyama F, Tsunada S, Fujimoto K (2005). T cell deficiency leads to liver carcinogenesis in Azoxymethane-treated rats. Soc Exp Biol Med.

[31] Logarto PA, Silva YR, Guerra SI, Iglesias BL (2001). Comparative study of the assay of *Artemia salina* L. and the estimate of the medium lethal dose (LD50 value) in mice, to determine oral acute toxicity of plant extracts. Phytomedicine.

[32] Khan MR, Zehra H (2013). Amelioration of CCl_4_-induced nephrotoxicity by *Oxalis corniculata* in rat. Exp Toxicol Pathol.

[33] Khan RA, Khan MR, Sahreen S (2012). Protective effects of rutin against potassium bromate induced nephrotoxicity in rats. BMC Complement Altern Med.

[34] Khan RA, Khan MR, Sahreen S (2012). CCl_4_-induced hepatotoxicity: protective effect of rutin on p53, CYP2E1 and the antioxidative status in rat. BMC Complement Altern Med.

[35] Sahreen S, Khan MR, Khan RA, Alkreathy HM (2015). Protective effects of Carissa opaca fruits against CCl4-induced oxidative kidney lipid peroxidation and trauma in rat. Food Nutr Res.

[36] Adewole SO, Salako AA, Doherty OW, Naicker T (2007). Effect of melatonin on carbon tetrachloride_induced kidney injury in Wistar rats. Afr J Biomed Res.

[37] Dogukan A, Akpolat N, Celiker H, Ilhan N, Bahcecioglu IH, Gunal AI (2003). Protective effect of interferon on carbon tetrachloride induced nephrotoxicity. J Nephrol.

[38] Srinivasan M, Rukkumani R, Sudheer AR, Menon VP (2005). Ferulic acid, a natural protector against carbon tetrachloride-induced toxicity. Fundam Clin Pharmacol.

[39] Khan MR, Siddique F (2012). Antioxidant effects of *Citharexylum spinosum* in CCl_4_ induced nephrotoxicity in rat. Exp Toxicol Pathol.

[40] Soni B, Visavadiya NP, Madamwar D (2008). Ameliorative action of cyanobacterial phycoerythrin on CCl_4_-induced toxicity in rats. Toxicology.

[41] Toyokuni S, Okamoto K, Yodoi J, Hiai H (1995). Persistent oxidative stress in cancer. FEBS Lett.

[42] Kawai Y, Nakao T, Kunimura N, Kohda Y, Gemba M (2006). Relationship of intracellular calcium and oxygen radicals to cisplatin-related renal cell injury. J Pharmacol Sci.

[43] Basnakian AG, Apostolov EO, Yin X, Napirei M, Mannherz HG, Shah SV (2005). Cisplatin nephrotoxicity is mediated by deoxyribonuclease I. J Am Soc Nephrol.

